# The Nef-Infectivity Enigma: Mechanisms of Enhanced Lentiviral Infection

**DOI:** 10.2174/157016211798842099

**Published:** 2011-10

**Authors:** Jolien Vermeire, Griet Vanbillemont, Wojciech Witkowski, Bruno Verhasselt

**Affiliations:** Department of Clinical Chemistry, Microbiology, and Immunology, Ghent University, Belgium

**Keywords:** Nef, HIV, infectivity, viral replication, mutation analysis, envelope protein, cholesterol, proteasome.

## Abstract

The Nef protein is an essential factor for lentiviral pathogenesis in humans and other simians. Despite a multitude of functions attributed to this protein, the exact role of Nef in disease progression remains unclear. One of its most intriguing functions is the ability of Nef to enhance the infectivity of viral particles. In this review we will discuss current insights in the mechanism of this well-known, yet poorly understood Nef effect. We will elaborate on effects of Nef, on both virion biogenesis and the early stage of the cellular infection, that might be involved in infectivity enhancement. In addition, we provide an overview of different HIV-1 Nef domains important for optimal infectivity and briefly discuss some possible sources of the frequent discrepancies in the field. Hereby we aim to contribute to a better understanding of this highly conserved and therapeutically attractive Nef function.

## 1 INTRODUCTION

Despite the globally declining number of new human immunodeficiency virus (HIV) infections [1] and the hopeful observation that cure from HIV infection does not seem impossible [2], the HIV pandemic still remains a very challenging opponent in the 21^st^ century. The ultimate solution would lie in the development of an effective and practically feasible therapy to eradicate HIV from infected patients, in combination with a safe vaccine to prevent new HIV infections. While waiting for these seemingly utopian breakthroughs, disease control mainly relies on the suppression of viral replication in HIV patients, by life-long treatment with antiretroviral therapy. However, the remarkable ability of the HIV virus to repetitively adapt and escape from antiretroviral agents, combined with toxic effects of these drugs, creates the need for a continuous development of new antiretrovirals. Therefore it is essential to keep exploring the HIV replication biology in search for new possible therapeutic targets. In this regard, the so-called “accessory” proteins of the HIV-1 virus (Nef, Vpr, Vpu, Vif) have received increasing attention [3, 4]. In contrast to the essential structural and enzymatic HIV proteins (Gag, Pol, Env), the accessory proteins are dispensable for viral replication in a number of *in vitro* systems. However, they are strongly conserved during *in vivo* infection and often appear to be crucial for viral pathogenesis (reviewed in [5]). One accessory protein for which there exists irrefutable evidence of its *in vivo* significance, is the 27-35kDa Nef protein. In this review we will focus on this extensively studied protein. In particular, we will discuss current insights in the well-known yet poorly understood ability of Nef to enhance the viral infectivity.

## 2 THE MULTIFACETED NEF PROTEIN

As early as 1991, infections of rhesus monkeys with *nef*-deleted simian immunodeficiency virus (SIV) revealed a dramatic reduction of viral loads and disease progression in absence of *nef *[6]. A couple of years later, the importance of HIV-1 Nef in pathogenesis of acquired immune deficiency syndrome (AIDS) was also established. Several groups reported the presence of deletions in the *nef* gene in a subpopulation of HIV infected patients with low viral loads and long-term asymptomatic survival [7-9]. Although some patients and monkeys infected with *nef*-deleted HIV [10, 11] or SIV [12] respectively, eventually progress to AIDS, Nef does seem to be indispensable for establishment of high viral loads and largely accelerates the disease progression. Combined with the fact that Nef expression alone can independently induce AIDS-like pathologies in mice, without the requirement of any other HIV components [13], this designated the protein as an attractive candidate for therapeutic targeting. This of course evoked a large scientific interest in unraveling the specific role that is reserved for Nef during the viral life cycle. Twenty years after its initial identification as an *in vivo *pathogenic factor, the number of known Nef effects is still increasing year by year. However their individual contribution to disease progression as well as their precise underlying molecular mechanisms are still largely unclear.

Like all accessory proteins, Nef contains no enzymatic activity nor does it serve a structural function. Instead, Nef turned out to be a master in rearranging the signaling and trafficking pathways of the infected host cell, in a way that is beneficial for the viral replication and spreading. Nef does this by physically interacting with specific cellular proteins involved in these pathways, through its multiple effector domains (reviewed in [14, 15]). Many different biological effects result from these interactions and they all seem to co-operate in a two-way strategy to promote the viral spread: firstly, by evading the anti-viral immune responses of the host and secondly, by directly enhancing the viral replication. The former is mainly based on the well-known capacity of Nef to modulate expression of receptors involved in immune response (e.g. MHC-I) on the surface of the infected cell. Consequently, the functional interaction between the infected cell and a non-infected immune cell is disturbed (reviewed in [16]).

The effect of Nef on viral replication itself, seems to be more complex. Initially, Nef was even considered to suppress HIV replication and to promote the establishment of latency (hence its name *negative regulatory factor*) [17]. Following some controversial *in vitro* results [18, 19] and the *in vivo* demonstration of the positive effect of Nef on viral load [6], it became clear that Nef does enhance HIV replication *in vitro*, especially in primary cells [20, 21]. In contrast to the *in vivo* situation, viral replication in these *in vitro* environments is not subjected to the influence of a complete host immune system. Therefore the enhanced replication, observed *in vitro*, can theoretically be attributed to one of the three following mechanisms: (1) It can result from an increase in the number of viral particles produced and released by infected cells; (2) The transmission of viruses from the virus producing cell to the target cell could be facilitated; (3) The intrinsic capability of the newly produced virions, to infect new target cells (the “viral infectivity”) could be enhanced. Over the years it became clear that Nef probably operates at all three levels. First, by subverting T cell signaling pathways and facilitating T cell activation, Nef enhances the activity of the HIV-1 LTR promoter, which in turn might promote the virion production [22-24]. Furthermore, Nef is thought to increase the release of viral particles by downmodulating the CD4 receptor on the virus producing cells [25]. Secondly, remodeling of the host actin cytoskeleton by Nef might promote the cell-to-cell transmission of virions, although this is still controversial [26, 27]. Also, in co-cultures of T cells and dendritic cells, Nef is thought to enhance the transmission of virus to T cells [28]. However, the most widely accepted of these three Nef functions, is the capacity of Nef to increase the infectivity of viral particles. Nevertheless, the mechanism behind this function remains largely obscure and new theories frequently arise in literature. In following sections we will discuss overall progress in understanding the molecular basis and functional mechanism of infectivity enhancement by Nef. Furthermore we will point out some possible causes of discrepancies in literature. For more in-depth discussion on the other possible mechanisms of viral replication enhancement by Nef, indicated above, we refer to an excellent review on the topic [16].

## 3 INFECTIVITY: WHAT’S IN A NAME?

In literature the term “infectivity” generally refers to the ability of a pathogen to cause infection of a host. Therefore it is often a combination of both its replicative and transmission capacities. However, when studying HIV infectivity and the contribution of Nef at cellular level, a slightly different interpretation is usually applied. This is based on the characteristics of the most commonly used assay to measure HIV infectivity *in vitro*: a HeLa-CD4 cell line that contains a Tat-responsive lacZ reporter is infected with equal amounts of cell-free virus and a single cycle of infection is allowed to occur before quantification of the amount of infected cells [29, 30]. In this setting, an increase in infectivity by Nef represents an increase in the intrinsic capability of a viral particle to productively infect a target cell, but is independent of effects of Nef on the production of viral particles or viral spread over the culture. Since most of the research on Nef and infectivity is based on this type of assays, we will use this interpretation throughout the review.

## 4 LESSONS ON NEF-MEDIATED INFECTIVITY ENHANCEMENT FROM MUTATIONAL STUDIES

The ability of Nef to enhance infectivity is a highly conserved property among different groups of primate lentiviruses. In a panel of 30 different Nef alleles from HIV-1 (groups M, N and O), HIV-2 and a large variety of SIV’s, Munch *et al*. demonstrated that more than 90% of them enhanced virion infectivity, although with a sometimes variable efficiency [31]. The fact that despite the genetic diversity, almost all Nef alleles enhanced infectivity, might indicate that different domains of the Nef protein attribute to this function. As mentioned before, Nef uses its different domains to interact with a large panel of cellular proteins. Therefore, identification of the domains involved in a particular Nef function, can shed light on the molecular mechanism of this function. Since the early nineties, a large number of Nef mutational studies have been performed in this regard. Some of these studies only focused on particular Nef functions and/or domains [32-49], whereas others analyzed a broad panel of isogenic mutants to obtain a better insight in the relation between different Nef activities and their contribution to optimal HIV replication [50-52]. An overview of the impact of different Nef mutations on infectivity enhancement, as observed in 22 different studies, is presented in Table **1**.

From studies using Nef-mutants deficient in association with the cellular membrane, it became clear that membrane-targeting is important for Nef enhanced infectivity. Both the G2A mutation, which abolishes the N-terminal myristoylation of Nef [32, 37, 39, 40, 46, 50-53] and mutations in a cluster of N-terminal basic residues (R4A4), which disrupts membrane targeting of Nef [36], resulted in an abundant decrease in infectivity. Furthermore, Giese *et al*. described the importance of two N-terminal lysine residues (K4/K7) for infectivity enhancement and showed that these residues are critical for incorporation of Nef in the lipid rafts [40]. Since these specialized microdomains in the plasma membrane are a preferred site for budding of HIV viral particles [54], these findings might indicate that the presence of Nef at the site of viral assembly is critical for its ability to enhance infectivity. Of note, viruses containing a Nef-mutant with highly increased lipid raft incorporation, by introduction of a palmitoylation site in the N-terminal *nef *region (G3C mutation), do not show a corresponding increase in infectivity [55, 56]. This lack of correlation between the K4/K7 loss-of-function [40] versus the G3C gain-of-function mutation [55, 56] can be interpreted as a saturation of the infectivity enhancement effect at physiological levels of Nef, since further enrichment of Nef in the lipid rafts does not further increase virion infectivity [55, 56].

Another Nef motif involved in infectivity enhancement is the polyproline stretch PxxP located in the core of the Nef protein [45, 46, 50-52]. This acidic region was demonstrated to bind with PACS-1 [57], the SH3-domain of the Src family kinases [58] and a complex of cellular proteins including Pak-2 [58, 59]. This might indicate that the ability of Nef to alter the cellular signaling pathways is important for its infectivity enhancing function. However, a direct contribution of these interaction partners is not demonstrated yet.

The dileucine motif in Nef (LL164/165 (*nl4-3* allele)) is also required for full infectivity of HIV-1 virions. This domain is known to mediate the interaction of Nef with the clathrin-coated vesicle-associated adaptor complexes (AP-1, AP-2, AP-3) and is therefore important for the well-known ability of Nef to enhance the internalization and modify the intracellular trafficking of membrane proteins [47, 60]. Therefore, alteration of membrane protein expression or a general disruption of protein trafficking might be required for enhancement of virion infectivity.

Also oligomerization of the Nef protein was proposed to be a determinant of infectivity as demonstrated in studies using mutations of two arginine residues at position 105 and 106 or the aspartic acid residue at position 123 (RR105/106AA and D123G respectively) [41, 43, 45, 51-53]. These residues are thought to form salt bridges necessary for the stabilization of Nef oligomerization [61, 62], although this model of oligomerization and the necessity of the D123 residue in this regard, was recently challenged by Kwak *et al*. [63].

## 5 MECHANISM OF INFECTIVITY ENHANCEMENT BY NEF

A well-known fact about Nef-mediated enhancement of infectivity, is that the infectivity of *nef*-deleted virions can be rescued completely by expressing Nef in the virus producing cell. However, if virions were produced in the absence of Nef, their infectivity cannot be rescued by expression of Nef in the target cells [30, 64, 65]. This shows that infectivity enhancement is not mediated by an action of the newly expressed Nef in the infected cell, but requires the presence of Nef during production of the viral particles. Therefore, Nef must somehow modify the virions during their biogenesis and thereby alter their behavior during subsequent infection of the target cells. Understanding the mechanism of Nef-mediated infectivity enhancement consequently consists of two questions: what is the nature of the modification imprinted by Nef in the virus producing cell and how is the infection process of the viruses affected by this modification?

### 5.1 Nef-Mediated Modification of Virion Particles in the Producer Cell

#### 5.1.1 Virion Incorporation of Nef

**Fig. (1):**
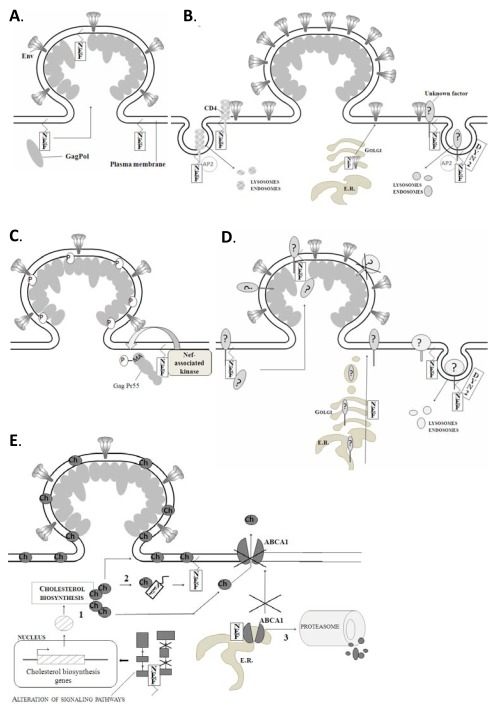
Nef-mediated modifications of the virions during virion biogenesis (at the site of viral assembly). Proposed modifications of nascent virions as a consequence of Nef expression in the virus producing cells are represented. (**A**) Expression of Nef during viral production results in incorporation of small amounts of Nef into the budding virion. (**B**) Nef enhances the incorporation of Env glycoproteins into the budding virion, by (left) a CD4-dependent mechanism: Nef downregulates CD4 and prevents sequestration of Env by CD4; by (right) CD4- independent mechanisms: Nef downregulates a yet unknown Env sequestering factor, other than CD4, in a Dyn-2 dependent way, or Nef enhances membrane trafficking of Env by reducing the retention of Env precursors. (**C**) Nef enhances phophorylation of matrix (MA) proteins, mediated by Nef-associated kinases. (**D**) Nef regulates the incorporation of yet unknown cellular factors into the budding virion, by (left) binding to cellular factors, resulting in their co-incorporation with Nef in the virion; by (right) regulating surface expression of cellular factors at the site of viral assembly, by affecting their trafficking to the surface membrane or by downregulating them from the cell surface in a Dyn2-dependent way. (**E**) Nef enhances the incorporation of cholesterol into the budding virion in different ways: (1) alteration of cellular signaling pathways by Nef results in increased expression of the cholesterol biosynthesis genes and thereby increased synthesis of cellular cholesterol; (2) Nef binds to newly produced cholesterol and promotes its transport to the sites of viral assembly; (3) Nef impairs the efflux of cellular cholesterol by stimulating proteosomal degradation of the cholesterol transporter ABCA1.

The most notable compositional difference between virions produced in the absence and presence of Nef, is the incorporation of small amounts of Nef itself in the latter [65, 66] (Fig. **1A**). It is therefore tempting to speculate that the increased infectivity of Nef-containing virions, results from a direct action of these virion-delivered Nef molecules during the early steps of infection. The fact that another virion-incorporated HIV-1 accessory protein, Vpr, is capable of enhancing viral infectivity (reviewed [67]) further supports such a hypothesis. However, over the years evidence accumulated against a role for virion Nef in infectivity enhancement.

As mentioned earlier, the infectivity of a genetically *nef*-deficient virus cannot be rescued by expression of Nef in the target cell [64, 66], rendering an infectivity enhancing activity of Nef in the target cell doubtful. Virion-incorpo-rated Nef is known to be cleaved at its WL_57-58_ motif by the viral protease during virion maturation [65, 66, 68]. It has therefore been suggested that *trans* complementation of Nef in the target cell might not provide the correct form of Nef [65]. This is however unlikely since HIV Nef mutants and SIV Nef proteins that lack the sequence required for protease cleavage, are still capable of enhancing viral infectivity [39].

Over the years multiple studies have investigated the virion incorporation of Nef mutants with a known defect in their ability to increase viral infectivity. These studies revealed the existence of both mutants that show efficient [38, 50] as well as mutants with inefficient [36, 50, 69] virion incorporation. However, correlation analysis does neither allow to exclude nor accept a role of virion Nef in infectivity enhancement, since incorporated mutants are not necessarily functional and non-incorporated mutants might also be defective for Nef activities in the producer cell that are required to enhance infectivity. Conversely, Fackler *et al*. showed that mutation of the Nef EEEE_66-69 _acidic stretch (SF2 *nef* allele) by alanine substitution results in loss of virion incorporation while maintaining the capacity to increase viral infectivity [50]. Since this indicates that infectivity enhancement can occur in absence of virion incorporated Nef, an active role of virion Nef in this regard would become unlikely. However, it has to be noted that the absence of this particular Nef mutant in the virion was questioned recently [70].

To avoid the use of Nef mutations with possible pleiotropic effects, two recent studies tried to manipulate virion incorporation of intact Nef by fusing it to proteins with known high incorporation levels in HIV-1 virions. Qi *et al*. showed that N-terminal fusion of Nef to the Gag interacting host protein Cyclophylin A (CypA), preserved its ability to enhance HIV-1 infectivity and resulted in high levels of the fusion protein in viral particles. Upon pharmacologic or genetic inhibition of the CypA-Gag interaction, virion incorporation was disrupted and this corresponded with a complete loss of infectivity enhancement. Although this study demonstrates a strong correlation between virion incorporation of Nef and infectivity enhancement, the authors cannot exclude that Nef acts prior to virion maturation and budding in the virus producing cell [70]. In this regard it is worth noting that N-terminal fusion will disrupt the myristoylation of Nef. Therefore the CypA-Gag interaction in this study might not only be required for virion incorporation, but also for association of Nef with the site of virion assembly. Another system to modulate virion incorporation of unmutated Nef proteins was described by Laguette *et al*. and involves C-terminal fusion of Nef with the virion incorporated HIV-1 Vpr protein. By introducing a viral protease cleavage site between Nef and Vpr, maturated virions contain high levels of the full-length Nef and Nef fragments that are found in wild-type HIV-1 virions. Nevertheless, these virions were only as infectious as virions produced in the absence of Nef. This indicates that an inhibitory effect of the Vpr sequence fused to Nef prevents its infectivity enhancing functions in the virus producing cell and that the presence of Nef in viral particles alone is therefore not sufficient to increase HIV infectivity [71].

#### 5.1.2 Virion Incorporation of Other Viral Proteins

Analogous to the concept of virion Nef levels discussed above, Nef might increase the virion infectivity by modulating the incorporation of other viral proteins in the budding virions. Over the years multiple studies failed to detect any differences in the composition of virions produced in the presence or absence of Nef, with regard to the amounts of genomic RNA, *gag*-derived structural proteins (CA, MA and NC), viral enzymes (RT and IN) and Vpr [30, 50, 56, 65, 72, 73].

On the other hand, levels of virion envelope glycoproteins were shown to be modestly or even highly reduced in *nef*-deleted compared to wild-type to HIV-1 virions, if these virions were produced in CD4+ expressing cells [74-77]. It is known that high cell surface levels of CD4 can largely decrease the infectivity of viral particles by sequestering the viral envelope proteins and preventing their incorporation in the nascent virions [75, 77]. Therefore, the well-described ability of Nef to downmodulate the CD4 receptor from the cell surface, is thought to alleviate this CD4-mediated blockage of Env incorporation in the virus producing cell [74-77]. This phenomenon is known in literature as the CD4 dependent mechanism of Nef-mediated infectivity (Fig. **1B**). Nevertheless, Nef can still increase virion infectivity if CD4 is not expressed in the virus producing cell [30, 32, 75]. Furthermore, the effect of Nef on CD4 and infectivity seems to be genetically separable, since certain Nef mutations were demonstrated to affect only one of the two functions [46, 52]. Therefore, Nef definitely employs CD4-independent mechanisms to enhance virion infectivity. In this regard, two more recent reports suggest that Nef can even increase the virion Env incorporation in a CD4-independent way [35, 78], possibly by reducing the retention of Env precursor proteins at the *cis*-Golgi [78]. Another mechanism proposed by Pizzato *et al*. is Nef-mediated downregulation of an unknown surface factor that sequesters Env [37] (Fig. **1B**) However, it has to be noted that a Nef-dependent increase of viral envelope levels is not observed by all groups, even if virions are derived from CD4 expressing cells [30, 56, 65, 73]. Although the underlying causes of these discrepancies are not clear, producer cell-type dependent differences might be involved, such as the surface levels of CD4 [74, 77]. When considering viruses produced by the physiologically more relevant primary CD4+ T cells, the extent of this Nef function still remains controversial, with studies reporting 600% to only 15% decrease of virion Env levels upon viral production in absence of Nef [35, 76]. Another confounding factor might therefore be the actual contribution of Nef to CD4 downmodulation at the moment of virus collection, since CD4 downmodulation is known to occur in a Nef-independent manner during later stages of infection, by actions of both Vpu and Env. Consequently, the corresponding inhibition of Env sequestration by CD4 would also become a Nef-independent phenomenon at this time point [35].

Alternatively to influencing the amounts of incorporated viral proteins, Nef could operate by regulating the post-translational modification of these proteins. Swingler *et al*. demonstrated that Nef strongly enhances the serine phosphorylation of the HIV-1 matrix (MA) proteins during the virion biogenesis and this modification is likely performed by Nef-associated cellular kinases [79] (Fig. **1C**). Furthermore, serine phosphorylation of MA seems to be especially important for optimal virion infectivity [80, 81]. However, Dorfman *et al*. demonstrated that Nef is still fully capable of enhancing viral infectivity when viruses lack the MA protein [82]. Therefore, the actual purpose of this Nef effect and its relevance for infectivity enhancement remains to be determined.

#### 5.1.3 Virion Incorporation of Cellular Proteins

In addition to viral proteins, virions contain a significant amount of host cellular proteins [83-85]. As mentioned before, HIV particles egress from the cell through specialized microdomains, called lipid-rafts, and thereby incorporate the cellular lipid raft-associated proteins (as reviewed in [83]). Furthermore, cellular proteins could be incorporated into the virion due to their presence at the site of viral assembly, for instance by an interaction with a viral protein (Fig. **1D**). Interestingly, HIV-1 infectivity is known to be enhanced by incorporated human leukocyte antigens (HLAs) [86, 87], costimulatory molecules (CD80 and CD86) [88], and intracellular adhesion molecule-1 (ICAM-1) [89, 90] probably because they facilitate the association of viral particles with the target cells. As mentioned by others [71], it is therefore tempting to assume that Nef enhances the virion infectivity by modifying the incorporation of cellular proteins. To our knowledge, there is currently no published data available on the cellular protein content of wild-type versus *nef*-deleted viral particles and therefore, evidence supporting this hypothesis is still lacking. However, different findings are in line with a mechanism for nef-mediated infectivity enhancement based on cellular protein incorporation.

As mentioned before, there’s a genetic correlation between the enhancement of viral infectivity by Nef and its ability to alter the intracellular trafficking and sorting of cellular membrane proteins [42, 47, 91-94]. Furthermore, Pizzato *et al*. demonstrated that both dynamin2 (Dyn2) and clathrin are absolutely required in the producer cell for Nef-mediated infectivity enhancement in a CD4-independent way [37]. Since both of these proteins are involved in clathrin-mediated endocytosis of membrane proteins, this strengthens the theory that Nef enhances virion infectivity by altering the membrane expression of cellular proteins in the producer cell. It is possible that Nef uses this mechanism to alter the protein composition of virions budding from these membranes (Fig. **1D**). Finally, as mentioned above, infectivity of HIV particles is enhanced by incorporation of ICAM-1 due to the binding with LFA-1 on the target cells [89, 90]. Recently, it was demonstrated that ICAM-1 expression is up-regulated by Nef in a vascular endothelial cell line [95], opening perspectives for a possible cellular target protein of Nef involved in infectivity. Therefore, studies analyzing the incorporation and exclusion of host proteins in virions produced from HIV-infected primary cells with or without Nef might reveal new insights in the mechanism of Nef enhanced infectivity.

#### 5.1.4 Lipid Composition of Viral Particles

As mentioned before, lipid rafts are a preferential site for budding of HIV viral particles and the Nef motifs that target Nef into these microdomains seem to be important for its infectivity enhancing function [40]. Furthermore, it was shown that disruption of these lipid rafts in the producer cell decrease the infectivity of Nef-containing viruses, while it has no effect on *nef*-deleted viruses [96]. These findings indicate that infectivity enhancement by Nef occurs at the site of virion budding. Lipid rafts are, next to their incorporation of particular cellular proteins, characterized by a specific lipid composition, which resembles the HIV viral particle lipid composition [97, 98]. Given the importance of host membrane and virion-associated lipids for maintenance of retroviral infectivity (reviewed in [54]), several groups investigated if Nef might alter the incorporation of certain lipid species into the cellular and viral membranes to optimize the viral infectivity.

In this regard Zheng *et al*. demonstrated increased levels of newly synthesized cholesterol in both cellular lipid rafts and virion particles when Nef was present in the producer cell [69]. This effect was attributed to two distinct mechanisms. First, Nef was shown to stimulate the cellular biosynthesis of cholesterol, by augmenting the expression of the cholesterol biosynthesis enzyme CYP51. Secondly, they identified a cholesterol binding motif in the C-terminal region of Nef, which was required for both the increased cholesterol incorporation and Nef-mediated infectivity enhancement of the viral particles. Therefore, Nef was proposed not only to increase synthesis, but also the transport of cholesterol to the lipid rafts, resulting in viral particles with an increased cholesterol content and a higher infectivity [69] (Fig. **1E**). Microarray analysis of HIV-1 infected CD4+ T cell lines by van ‘t Wout *et al*., subsequently revealed an increased expression of multiple additional genes involved in cholesterol biosynthesis and uptake, during infection. Interestingly, these changes were only observed with viruses carrying a functional Nef gene [99]. However, we could not confirm the increased expression of CYP51 or any of the cholesterol pathway genes reported by van ‘t Wout *et al*., in a microarray study of Nef-expressing primary CD4+ T cells (unpublished data). An additional mechanism applied by Nef to increase virion cholesterol content might be the reduction of cholesterol export from infected cells (Fig. **1E**). Mujawar *et al*. demonstrated that Nef impairs the efflux of cholesterol from monocyte-derived macrophages (MDM’s) by targeting the cholesterol transporter ABCA1 and this is associated with intracellular lipid accumulation [100]. A similar phenomenon was recently demonstrated by the same group in primary CD4+ T cells (PBL’s) [101]. Furthermore, when ABCA1 cholesterol efflux was artificially stimulated during viral production, virions obtained from both MDM’s and PBL’s had a lower cholesterol content and an equally decreased infectivity. This indeed suggests a necessity for limiting cholesterol efflux during viral production in order to ensure adequate viral infectivity [100, 101], However, a direct correlation between this Nef function and its capacity to increase virion infectivity has not been demonstrated yet.

Although Nef seems to affect cellular cholesterol metabolism in multiple ways, the actual increase of virion cholesterol levels by Nef is not universally accepted. A lipidome analysis of HIV-1 virions produced in the presence or absence of Nef, could not confirm the effect of Nef on cholesterol content, originally observed by Zheng *et al*, but did observe other Nef-mediated alterations in virion lipid composition, such as enrichment of sphingomyelin [56]. With regard to cholesterol analysis, this study differed from the one of Zheng *et al*. by performing quantification of the total levels of virion cholesterol [56] as opposed to newly synthesized cholesterol only [69]. It might be possible that a modest increase in newly synthesized cholesterol incorporation is not reflected in the total cholesterol levels. However, analysis of virions produced from non T-cell lines, performed by another group, did show a small increase in total cholesterol levels by Nef, although this was not statistically significant and did not fully correspond with the level of infectivity enhancement [101]. The different outcomes in these three studies might therefore also be attributed to producer cell dependent differences, such as their expression levels of ABCA1 [69, 101].

It is quite clear that host membrane and virion-associated cholesterol are indispensable for optimal viral infectivity and this seems particularly the case for viruses produced in the presence of Nef [69, 101, 102]. This supports the idea that Nef-mediated enhancement of the viral infectivity occurs through a lipid raft-dependent mechanism in the virus producing cells. However, it is still controversial if an alteration of the virion lipid composition by Nef takes part in this process. Such a modification is anyhow unlikely to fully account for the effect of Nef on viral particle infectivity, since it is possible to demonstrate a loss of Nef-mediated infectivity enhancement, without observing changes in the viral lipid composition [37, 56].

### 5.2 Impact of Virion Modification on Infection Events in the Target Cell

The next question in the Nef infectivity enigma, concerns the actual consequence of virion modification by Nef in the producer cell, during subsequent infection of the target cell. In other words, how do virions, produced in the presence of Nef, manage to achieve a higher success-rate of productive infection? As mentioned before, the absence of Nef during viral production cannot be complemented by expression of Nef in the target cell. Therefore, the answer to this question most likely involves an increased efficiency of a certain step during infection, preceding the transcription of viral genes.

#### 5.2.1 Impact of CD4-Dependent Virion Modifications on Infection Events

**Fig. (2):**
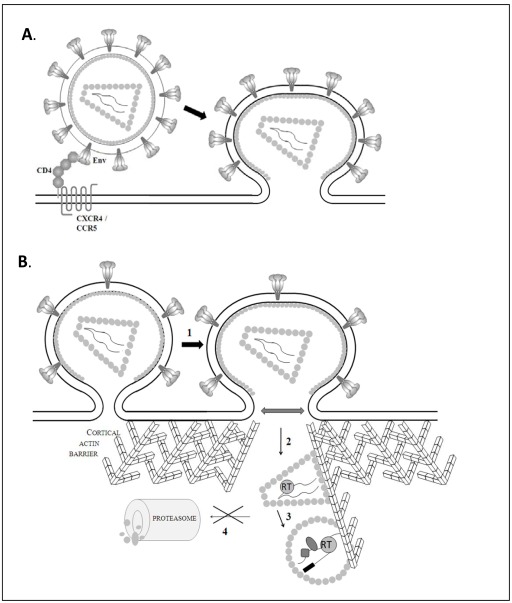
Mechanisms of enhanced infection of the target cell by Nef-modified virions. Proposed steps of the viral infection process that are facilitated due to Nef-mediated modifications of the virion. (**A**) CD4-dependent virion modifications: higher levels of Env glycoproteins in the virions results in an increase of functional Env-CD4 receptor interactions and thereby increase of the likelihood of productive infection. (**B**) CD4-independent virion modifications: a yet controversial modification imprinted by Nef during virion production (1) facilitates the dilatation of the fusion pore and consequently the cytoplasmatic delivery of the virion core; (2) facilitates movement of the virion core through the cortical actin barrier; (3) facilitates activation of the reverse transcription complex; (4) protects the virion core against proteosomal degradation.

HIV infection is initiated by binding of the viral envelope glycoproteins to the CD4 receptor and CXCR4/CCR5 co-receptor on the target cell. As mentioned before, Nef is thought to enhance the levels of envelope proteins incorporated in the virion, especially if they are produced in CD4 expressing cells. Therefore, the CD4-dependent mechanism of Nef-mediated infectivity enhancement probably relies on increasing the number of functional envelope-CD4 receptor interactions and thereby the likelihood of productive infection (Fig. **2A**). Artificial modulation of the virion envelope levels has indeed shown that the number of productively infected cells in the culture increases, when gradually increasing the amount of incorporated Env proteins in the virion [103]. Alternatively, target cells expressing higher levels of CD4 are more prone to productive infection, compared to cells with a lower surface density of the HIV receptor [74, 77, 103]. In this regard it has been demonstrated that the differences in infectivity between Nef-deleted and wild-type virions are more pronounced upon infection of cells that express lower amounts of CD4. Therefore, the lower amounts of Env proteins in the Nef-deleted virions might indeed hamper a successful interaction with the CD4 receptor and this phenomenon can be overcome by high levels of CD4 on the target cell [74, 77].

Of note, two early studies failed to detect a difference in the cell surface binding capacities of *nef*-deleted and wild-type virions [73, 104]. However, one of these studies observed equal amounts of Env proteins incorporated in both types of viruses [73]. This again demonstrates that the effect of Nef on Env incorporation and therefore interaction with the target cell CD4-receptor, is probably dependent on the producer-cell and the time point of analysis.

#### 5.2.2 Impact of CD4-Independent Virion Modifications on Infection Events

Nef also enhances the infectivity of viruses produced in cells that do not express CD4. Just like the virion modification responsible for this effect, the step of the infection process that is affected by this modification, remains elusive. However, over the years some enlightening observations have been made.

First, the increase in infectivity is already detectable at the stage of reverse transcription: infection with a *nef*-deleted virus results in a significantly lower accumulation of both early and late reverse transcription products in the cells. However, purified *nef*-deleted and wild-type viruses show an equal capability to complete the reverse transcription of their genome in a cell-free environment. Therefore the reverse transcriptase activity itself is not hampered when virions are produced in the absence of Nef [64, 73, 104]. Secondly, three different groups demonstrated that the fusion process between the viral and cellular membrane was equally efficient for *nef*-deleted en wild-type viruses [105-107]. By contrast, larger amounts of viral capsid proteins were detected in the cytoplasm, very soon after infection with viruses produced in the presence of Nef [108]. The latter observation can be interpreted as either an increased cytoplasmic delivery of the virion core or an increased efficiency of the virion uncoating process [107, 108]. Based on these observations, the infectivity advantage of virions produced in the presence of Nef, seems to result from an increased efficiency of a post-fusion event that occurs before completion of reverse transcription.

More insight in the nature of this event, can be obtained from particular experimental conditions that were able to restore the infectivity defect of *nef*-deleted viruses. These have led to different theories regarding the specific stage of HIV-1 infection that is favored by Nef-induced virion modifications (Fig. **2B**)

##### 5.2.2.1 Facilitation of Virion Core Movement Through the Cortical Actin Barrier

Soon after the discovery of the effect of Nef on infectivity, it was shown that pseudotyping HIV with the vesicular stomatitis virus envelope glycoprotein (VSV-G) overcomes the requirement of Nef in the virus producing cell [109, 110]. Conversely, viruses pseudotyped with amphotropic murine leukemia virus (MuLV) envelope remain dependent on Nef for optimal infectivity [30, 64]. While the latter modification preserves normal fusion of the viral membrane at the cell surface, the VSV-G envelope targets virions for cellular entry via the endocytotic pathway and promotes fusion upon endosomal acidification. This indicates that the Nef-dependent stage of viral infection can be bypassed by a deviation from the normal route of viral entry [109-112]. A first hypothesis about the identity of this early post-fusion infection stage, was provided by Campbell *et al*. They demonstrated that disruption of the cortical actin cytoskeleton in target cells could also replace the need for Nef. Since this dense layer of actin just beneath the plasma membrane is known to obstruct the infection of several pathogens, Nef-induced virion modifications might facilitate the movement of the virion core through this cortical actin barrier. This stage of infection would however be bypassed when virions are targeted for entry through the endosomal pathway, which explains the redundancy of Nef for viruses pseudotyped with a pH-dependent envelope, such as VSV-G [105]. In support of this hypothesis, it was recently demonstrated that the cortical actin network indeed poses a major postentry barrier to HIV-1 infection of resting CD4+ T cells [113].

Accumulating evidence however suggest that HIV-1 might also infect cells by fusing with the endosomal membranes, following endocytotic uptake [114, 115]. Although the contribution of this pathway to HIV entry of T cells is still controversial [116, 117], it appears to be a very prominent entry route in HeLa-CD4 cells [115], which are the most commonly used cell lines for HIV infectivity assays. Furthermore, Pizzato *et al*. recently reported an ability of Nef to enhance infectivity when entry occurs through an endosomal pH-dependent route [112]. Therefore, it is likely that *nef*-deleted viruses encounter other obstacles during the infection process, in addition to the crossing of the cortical actin layer.

##### 5.2.2.2 Facilitation of the Reverse Transcription Complex (RTC) Activation

An alternative virion modification that rescues the infectivity phenotype of the *nef*-deleted virus was demonstrated by Khan *et al*. They showed that the presence of Nef in the producer cell is not required when reverse transcription is artificially initiated in the virion prior to infection [72]. As mentioned before, wild type and *nef*-deleted virions show equal reverse transcriptase activity in a cell-free environment [64, 72, 73, 104]. Therefore, this observation points towards a specific block in the infection process of the *nef*-deleted viruses accompanying the stage of reverse transcription activation. Unfortunately, current knowledge about the time point and the specific event that triggers RT activation are quite limited. Initiation of reverse transcription seems to require an interaction of the RTC with the actin cytoskeleton [118], indicating that intracellular trafficking of this complex might be necessary for activation. Therefore it was suggested that a Nef-induced virion modification might guide the RTC to a specific cellular compartment that provides an optimal environment for RT activation, such as an adequate nucleotide concentration. Alternatively, this virion modification might be necessary to recruit one of the many cellular factors involved in the intracellular HIV reverse transcription process (reviewed in [119]).

##### 5.2.2.3 Protection Against Proteasomal Degradation

During early stages of viral infection, the cellular proteasome is thought to behave as an anti-viral defense mechanism by degrading viral cores upon cytoplasmic entry. This was suggested by different reports that observed an increase in HIV-1 infection when target cells were treated with proteasome inhibitors during the first hours of infection [120, 121]. Recently, Qi *et al*. demonstrated that this type of treatment preferentially enhanced the infectivity of *nef*-deleted viruses and therefore results in a large decrease of the normal infectivity advantage of Nef-containing viruses. This might indicate that Nef-induced virion modifications are necessary to render viral particles less susceptible for virion core degradation. A possibility, proposed by Qi *et al*., is a specific inhibition of virion protein ubiquitylation by Nef in the producer cell, which would limit recognition of the virion core by the proteasome complex in the target cell. Alternatively and in analogy with a Nef-dependent guidance of the RTC, a Nef-induced modification might allow correct trafficking of the virion core into a productive infection pathway, thereby bypassing a proteasomal cellular compartment that would cause abortive infection [70, 122].

##### 5.2.2.4 Facilitation of fusion pore dilatation

Fusion between the viral and cellular membrane results in formation of an initial small fusion pore, which requires subsequent enlargement to permit passage of the virion core [123, 124]. The theory that Nef-induced virion modifications might assist in the latter process, came from the observation that larger amounts of capsid proteins are detectable in the cytoplasm after infection with wild-type viruses compared to *nef*-deleted viruses. Therefore, Nef was thought to specifically promote the infection steps that regulate cytoplasmic virion delivery [108]. Since *nef*-deleted viruses are not hampered in their ability to mediate membrane fusion [105-107], a facilitation of the fusion pore dilatation by the Nef-induced modifications, would be consistent with this [106, 112]. As pointed out by others [112], Nef-mediated assistance at this particular infection stage is not that unlikely, since pore dilatation is known to be a very energy-demanding process and therefore often a limiting step in productive viral infection [125]. However, the higher levels of cytoplasmatic viral capsid proteins during wild-type virus infection, observed by Schaeffer *et al*. [108], can be interpreted in different ways. The particular technique used in this study to isolate cytosolic cell fractions, might eliminate *nef*-deleted virion cores that were stuck in the cortical actin barrier [105, 106] or might preferentially detect the free viral capsid proteins that are released during virion uncoating [107].

Despite many theories about the Nef-dependent stage in early HIV infection, a solid model explaining all of the above observations is still lacking. A Nef-induced virion modification seems to facilitate an early post-fusion event and different groups suggested a role in intracellular trafficking of early viral complexes. Identification of the nature of this event is however hampered by the limited knowledge regarding the intracellular localization and order of the early HIV infection processes. Furthermore, HIV infection pathways seem to be cell-type dependent and HeLa-CD4 cells might not always prove a relevant model to study these particular events [116, 117]. It will probably require identification of the responsible virion modification, to fully characterize the Nef-dependent infection step.

## 6 IMPORTANCE OF NEF-MEDIATED INFECTIVITY ENHANCEMENT FOR HIV REPLICATION AND PATHOGENESIS

Since the identification of Nef as a virulence factor, an important question has been the actual contribution of the many Nef activities to viral replication and disease progression. A multitude of studies has addressed this by evaluating the replicative (and pathogenic) potential of HIV and SIV variants with differential activity of specific Nef functions, in primary cell cultures or macaques (reviewed in [126]). The importance of the infectivity enhancement function of Nef for viral replication and pathogenesis remains however controversial. Upon discovery of Nef’s effect on virion infectivity, this function was thought to be responsible for the increase in viral replication kinetics by Nef, observed in cultures of primary CD4+ T cells. It was suggested that the enhanced infectivity observed in a single-round of infection is subsequently potentiated over many viral life cycles and would in this way evoke in a major increase of the overall growth rates of the viruses [20, 21]. However, the actual difference in viral replication kinetics between *nef*-deleted and wild-type viruses, are much less spectacular then would be expected if such an amplification effect was occurring [26, 64]. Furthermore, different studies even failed to find a correlation between the extent of infectivity enhancement by different patient-derived or specifically mutated Nef alleles and their effect on viral replication in primary CD4+ T cells or human lymphoid tissue [33, 50, 52], as *nef* alleles deficient in their ability to enhance infectivity, were still fully capable to enhance viral replication [52]. In this regard Haller *et al*. suggested that the high efficacy of viral cell-to-cell transmission, which is the predominant mode of viral spread in T-cell cultures, might override differences in infectivity imprinted by Nef. Since viral cell-to-cell transmission is likely also important *in vivo*, this would question the relevance of the infectivity enhancement of Nef for HIV pathogenesis [26, 31]. However, a study in macaques indicates that this Nef function does contribute to efficient viral spread *in vivo*. Infection with an SIV variant that contained a *nef* allele with an intermediate infectivity enhancement function resulted in higher viral loads, compared to infection with a variant containing a *nef* allele with complete loss of this function, but equal activity in other Nef functions [127, 128]. Furthermore, *nef* alleles derived from macaques with higher viral loads were more active in enhancing viral infectivity in an *in vitro* assay, then *nef* alleles from macaques with low viral loads and attenuated disease [127]. Another argument for *in vivo* importance of Nef-mediated infectivity enhancement is the high level of conservation of this particular Nef function among different groups of primate lentiviruses [31]. This indicates the presence of selective pressure *in vivo* to maintain this Nef function. Of note, Pizzato *et al*. recently demonstrated that the glycogag protein of murine leukemia viruses is required for optimal infectivity of these viruses and can also rescue the infectivity of *nef*-deleted HIV. Since both proteins seem genetically unrelated, they might have independently acquired an infectivity enhancing function during evolution, indicating an important role in retroviral biology [129].

## 7 UNDERLYING CAUSES OF DISCREPANCY IN INFECTIVITY STUDIES

As indicated throughout this review, findings on Nef-mediated infectivity are often inconsistent across different studies, causing confusion about their relevance in the field. This is further illustrated by the widely divergent effects of Nef domain mutations on infectivity enhancement, as listed in Table **1**. As mentioned before, differences in the producer cell, such as CD4 expression levels, might contribute to these discrepancies. However, most of the studies discussed here focused on the CD4-independent effects of Nef on infectivity and used the same CD4 negative embryonic kidney derived cell line, 293T, for viral production. An important cause of discrepant results as inferred from Table **1**, is the use of *nef *alleles derived from different HIV-1 strains. A striking example in this regard is the Nef G2A mutation, which is known to completely abolish the infectivity enhancing function of most *nef* alleles, but causes only a moderate decrease in this function for the SF2 *nef *allele [50]. Furthermore, it is known that the dependency on Nef for optimal virion infectivity, can be affected by other HIV proteins [103]. Therefore, results will likely differ when analyzing *nef* alleles in context of their original genetic background or when introducing them in the genetic background of another HIV strain. Finally, observed effects on infectivity seem highly dependent on the experimental assay used to measure infectivity. Two well-known cell lines for single-cycle infectivity determination are the P4-CCR5 and TZM-bl indicator cells. Both of them were originally derived from the HeLa cell line and contain an integrated copy of HIV-1 long terminal repeat (LTR) linked to a β-galactosidase (P4-CCR5 and TZM-bl) and luciferase gene (TZM-bl) [29, 130]. An important difference between the two is that TZM-bl cells express much higher levels of CD4 but lower levels of CXCR4. Furthermore, TZM-bl cells are known to be more susceptible to HIV-1 infection, but the observed magnitude of Nef-mediated infectivity enhancement with this cell-line is often lower [31, 34]. In this regard, Schindler *et al*. reported that the F191R Nef mutation affected infectivity enhancement when measured in P4-CCR5 cells, but not in the TZM-bl cells [34]. However, it might be more relevant to investigate the effect of Nef on virion infectivity in primary target cells, instead of these indicator non T-cell lines. Different methods applied up till now are (1) determination of the % of infected cells 3 to 5 days after infection, by either the use of reporter viruses or intracellular p24 staining [34, 35], or (2) determination of the TCID50 [20, 44]. However, it might be difficult to separate effects of Nef on infectivity and replication in general, when looking more than 36 h post-infection. Single-cycle assays in primary cells would overcome this problem, but due to the low infection efficiency of cell-free virus, quantification of subtle infectivity differences would prove quite difficult at such an early time-point.

## CONCLUSION

Almost two decades after the discovery of Nef’s ability to enhance virus infectivity, the molecular mechanism of this function is still elusive. Identification of the virion modification imprinted by Nef in the producer cell is probably a prerequisite for understanding the behavioral difference of wild type and *nef*-deleted viruses in the target cell. Clarification of both parts of the *nef*-infectivity enigma could be hampered by the predominant use of biologically less relevant cell types. Viral production in CD4 negative producer cell lines is often necessary to study CD4-independent mechanism of Nef-mediated infectivity enhancement, but the mechanism and extent of virion modification might prove different in primary producer cells. Also, HIV infection pathways in the HeLa-CD4 target cells might differ from those in primary target cells. The relevance of Nef’s infectivity enhancing function has been questioned by lack of correlation with its effect on viral replication. However, the high level of conservation among different *nef* alleles indicates the importance of this function for *in vivo *HIV biology and it can therefore be considered as a possible therapeutic target.

## Figures and Tables

**Table 1.** Overview of Nef Domain Mutations Reported to Affect Infectivity Enhancement. Different Nef Domains are Separated by Alternating Shading

Mutation	HIV-Strain	Infectivity Assay				Function of Nef Mutated Region	References
		P4-R5 Assay	TZM-bl Assay	Prim. Cells	Mouse Model		
G2A	NL4.3, SF2, R8, HXB, LAI	0	0/+			Myristoylation signal	[32, 37, 38, 40, 46, 50-53]
Δ4-7	HXB Nef +	0				Incorporation lipid rafts	[46]
K4/7A	SF2		0			Incorporation lipid rafts	[40]
Δ12-39	SF2		0			Nef phosphorylation - Tat transactivation (NAKC)	[40, 50]
R17/19/21/22A	SF2, NL4.3	0	++			Membrane targeting	[36, 40, 50]
RER17-19AAA	R8	0				Membrane targeting	[52]
M20L	NL4.3	++			++	MHC1 ↓	[48, 53]
V30A	NL4.3	0					[51]
RD35/36AA	NL4.3, R8	+				CD4 ↓	[52]
L37Q	NL4.3			++ ^#^		CD4 ↓	[35]
C55A	R8	+				Protease cleavage site	[52]
CAW55-57LLL	HXB Nef +	0				Protease cleavage site - CD4 ↓	[38]
W57A	NL4.3, R9, HXB	0/+				Protease cleavage site - CD4 ↓	[51, 38, 39]
L58A	R9	+				Protease cleavage site - CD4 ↓	[39]
WL57/58AA	NL4.3, R8, R9, HXB	0/+/++		+ ^†^		Protease cleavage site - CD4 ↓	[38, 39, 44]
Δ59-61	SF2		0			CD4 ↓	[50]
W61A	SF2		+			CD4 ↓	[50]
Δ60-71	HXB Nef +	0				Binding PACS-1 - MHC1 ↓	[46]
E62-65A; E66-69A	NL4.3, SF2	+	+	++ ^†^		Binding PACS-1 - MHC1 ↓	[49]
E62/63Q	R8	+				Binding PACS-1 - MHC1 ↓	[52]
E63A	NL4.3	+				Binding PACS-1 - MHC1 ↓	[51]
E64/65Q	R8	+				Binding PACS-1 - MHC1 ↓	[52]
P69A	NL4.3	+				Binding Pak2 and SH3 domains	[51]
P69/72/75/78A	NL4.3, R8, HXB	0		+ ^†^	++	Binding Pak2 and SH3 domains	[44, 46, 52, 53]
P69/72A	NL4.3, R8	+/0				Binding Pak2 and SH3 domains	[52]
P72/75A, P76/79A	LAI, SF2	+	+			Binding Pak2 and SH3 domains	[37, 50]
P73A	SF2	0	+			Binding TCR zeta and Pak2	[45]
P75/78A	NL4.3, R8	++/0				Binding Pak2 and SH3 domains	[50, 51, 52]
P76A	SF2	0				Binding Pak2 and SH3 domains	[45]
RK77/82AA	NL4.3	+				Binding Pak2 and SH3 domains	[51]
V78A	SF2		+			Binding Pak2 and SH3 domains	[50]
P78L	NL4.3			+ ^#^		Binding Pak2 and SH3 domains	[35]
P79A	SF2	0				Binding Pak2 and SH3 domains	[45]
T80A	NL4.3, R8, HXB	0	+		++	PKC phosphorylation site	[46, 52, 53]
R81A	SF2		+			Binding Pak2 and SH3 domains	[50]
D86A/H89A	R8	0					[52]
R105/106L; R109/110L	NL4.3, SF2		+		++	Multimerization - binding Pak 2	[50, 53]
R105/106A	NL4.3, R8	+/0/0				Multimerization - binding Pak 2	[45, 51, 52]
R105Q	isolate sequence^*^	+				Multimerization - binding Pak 2	[43]
R106A	NL4.3, isolate sequence^*^	++/0				Multimerization - binding Pak 2	[43]
R106Q	isolate sequence^*^	0				Multimerization - binding Pak 2	[43]
D108A/D111A	R8	0				Binding thioesterase	[52]
D111G	NL4.3	0					[51]
L112A, L116A	LAI, SF2	0	+			Multimerization - binding Pak 2	[37, 50]
W113A	NL4.3	+					[51]
G119L	NL4.3	0					[51]
F121A	LAI	0				Binding thioesterase - dynamin2	[37]
D123A	LAI	0				Binding thioesterase - dynamin2 - multimerization	[37]
D123E	isolate sequence^*^	0				Binding thioesterase - dynamin2 - multimerization	[43]
D123G	NL4.3, LAI	+/0				Binding thioesterase - dynamin2 - multimerization	[41]
P136A	NL4.3	0					[51]
W141A	NL4.3	+					[51]
P147A	NL4.3	+					[51]
P147/150A	NL4.3	++			++		[51, 53]
E155Q	NL4.3, R8, LAI	++				Beta COP binding	[37, 51, 52]
K158E/E160A	NL4.3, R8	++				Binding AP-1/2/3	[51, 52]
L164/165A; L168/169A	NL4.3, R8, LAI, SF2	0/+	+	0 ^†^		Binding AP-1/2/3 and V1H - CD4 ↓ - sorting signal	[42, 44, 47, 50, 51, 52]
D174/175A; ED178/179AA	NL4.3, R8, SF2	+	+	0 ^†^		Binding AP-1/2/3 and V1H - CD4 ↓ - sorting signal	[50, 51, 52]
D175A	NL4.3	+				Binding AP-1/2/3 and V1H - CD4 ↓ - sorting signal	[51]
E177K	NL4.3			++ ^#^		Binding cRaf1 kinase - CD4 ↓	[35]
ERE177-179AAA	NL4.3, R8	+				Binding cRaf1 kinase - CD4 ↓	[51, 52]
E179A	NL4.3	+				Binding cRaf1 kinase - CD4 ↓	[51]
W183A	NL4.3	0					[51]
L189A	NL4.3	0					[51]
F191H	NA7, Pex^**^	+	++	++ ^‡^		Binding Pak2	[34]
F191R	NA7, Pex^**^	0	++	++ ^‡^		Binding Pak2	[34]
H193A	NL4.3	+				Binding Pak2	[51]

++ indicates activity comparable to the wt protein, + intermediate activity and 0 designates loss of function.

# viral infectivity calculated as percentage of p24+ cells 5 days post-infection;

† infectivity calculated as TCID50

‡ infectivity calculated as % GFP positive cells 3 days post-infection.

* DNA encoding Nef based on accession numbers AAA87489 and AAD48628.

** Pex: consensus nef allele based on nef sequences derived from 91 HIV-1 infected individuals at different stages of disease (ref [34]).

Remark: mutations only studied once and no effect on infectivity found in Hela or TZM-bl assay: Δ8-15 for HXB2 Nef allele; A56D for NA7 Nef allele; K201A/E204A for R8 Nef allele; P82A, R105A, R106K, R4A4 (R17/19/21/22A), E181Q for SF2 Nef allele and K7A, W13A, R17A, M20A, E24A, D36A, E38A, T44A, N51A, V74R, R77A, K82A, D86A, F90A, K94A, L100A, N126A, P131A, H166A, H171A, D174G, H199A for NL4.3 Nef allele (ref [43, 45, 46, 48, 50, 51]).
